# Characteristics of BDS Signal-in-Space User Ranging Errors and Their Effect on Advanced Receiver Autonomous Integrity Monitoring Performance

**DOI:** 10.3390/s18124475

**Published:** 2018-12-18

**Authors:** Zhipeng Wang, Wei Shao, Rui Li, Dan Song, Tinglin Li

**Affiliations:** School of Electronic and Information Engineering, Beihang University, Beijing 100191, China; wangzhipeng@buaa.edu.cn (Z.W.); shaowei@buaa.edu.cn (W.S.); songdan0207@163.com (D.S.); leetl@buaa.edu.cn (T.L.)

**Keywords:** ARAIM, signal-in-space user range errors, false alert probability, availability

## Abstract

Signal-In-Space User Range Errors (SIS UREs) are assumed to be overbounded by a normal distribution with a standard deviation represented by the User Range Accuracy (URA). The BeiDou Navigation Satellite System (BDS) broadcast URA is not compatible with the historical SIS URE performance that affects the Advanced Receiver Autonomous Integrity Monitoring (ARAIM) False Alert Probability (Pfa) and availability evaluation. This study compares the BDS broadcast and precise ephemeris from 1 March 2013 to 1 March 2017 to obtain SIS UREs. Through analyzing the statistical characteristics of the SIS UREs, we obtain the standard deviation *σ_URE_* for the accuracy and continuity and *σ_URA_* used for the integrity of the SIS UREs. The results show that the broadcast *σ_URA_* of 2 m cannot completely overbound SIS UREs for all BDS satellites, but the *σ_URA_* of 2.4 m can. Then, we use the *σ_URA_* of 2.4 m to evaluate the ARAIM Pfa and availability. The results show that the Pfa may increase to 2 × 10^−5^ and exceed its limit by an order of magnitude. We also consider the differences between the SIS UREs of Geostationary Earth Orbit (GEO), Inclined Geo-Synchronous Orbit (IGSO), and Medium Earth Orbit (MEO). The results indicate that all Pfa values calculated by the computed *σ_URE_* are less than the Pfa in the Integrity Support Message (ISM) for the worst-performing GEO satellite. The approximately 55% Pfa calculated by the computed *σ_URE_* is less than the Pfa in ISM for the worst-performing IGSO satellite. Most Pfa values calculated by the computed *σ_URE_* is less than the Pfa in the ISM for the worst-performing MEO satellite. For BDS satellites, the Pfa is mainly affected by *σ_URE_*. When the *σ_URA_* of 2.4 m is used to evaluate the availability, the computed availability is lower than the availability calculated by the broadcast *σ_URA_*/*σ_URE_* and the greatest degradation can reach 25%.

## 1. Introduction

Advanced Receiver Autonomous Integrity Monitoring (ARAIM) can provide global users with integrity monitoring service for vertical guidance [1]. The ARAIM algorithm is affected by several error sources. These errors can be divided into errors that arise in navigation satellites, affect signals during propagation, and emerge from the receiver or its surrounding environment [2]. This paper focus on analyzing the errors that arise in navigation satellites, such errors are called Signal-In-Space Errors (SISEs). 

SISEs are derived by comparing the broadcast ephemeris with the precise ephemeris that includes satellite orbit and clock errors, signal generation errors and antenna characteristics. The satellite orbit and clock errors are the largest sources of SISEs because the broadcast satellite location and clock states are inaccurate. Satellite orbit and clock errors constitute SIS User Range Errors (UREs). Therefore, SISEs can be described by SIS UREs [3].

For ARAIM users, the parameters in the Integrity Support Message (ISM) are important to evaluate the availability. The User Range Accuracy (URA) and URE are important parameters in the ISM. The value of the URA represents the standard deviation of the clock and ephemeris error used for integrity. The value of URE represents the standard deviation of the clock and ephemeris error used for accuracy and continuity [4]. These parameters should describe the characteristics of historical SIS UREs. Otherwise, these parameters may provide the misleading information to ARAIM users and affect the ARAIM performance. The URA and URE may also affect the ARAIM False Alert Probability (Pfa), which is the continuity budget allocated to disruptions due to false alerts, and has a value of 4 × 10^−6^ [5]. Therefore, the SIS UREs of constellation should be observed and analyzed.

Multiple scholars have studied SIS UREs for various constellations. Heng et al. at Stanford University studied GPS historical data between 2009 and 2011. Their results show that the SIS UREs characteristically vary among different GPS satellites. The SIS UREs of different satellites are slightly correlated, but still meets ARAIM’s requirement [6]. Santiago et al. at the German Aerospace Center analyzed GPS data for seven years as well as Galileo data for three months, and the results show that although the satellites employ the same model for the same constellation, different satellites perform differently, and the URA in the ISM should use different values for different satellites to account for the diversity of satellite performance [7]. Kazuma et al. at Stanford University analyzed GLONASS historical data between 2009 and 2016. The results show that the satellite failure rate dropped below 10^−4^, but the constellation failure rate did not meet the corresponding commitment over the eight years studied [8]. Walter et al. at Stanford University analyzed GPS historical data and GLONASS historical data between 2013 and 2016. The results show that GPS performance is consistent with its official parameters and that position error bounds formed from individual satellite ranging error bounds can bound any resulting positioning error. The GLONASS performance is consistent with its official parameters [9]. Zhang et al. analyzed data from the BDS C01-C12 satellites (except C02) for one month. The results show that the average SIS UREs for the Geostationary Earth Orbit (GEO), Inclined Geo-Synchronous Orbit (IGSO), and Medium Earth Orbit (MEO) satellites are different [10]. Most of these studies have analyzed the observed historical data of different constellations to describe the characteristics and accuracy of SIS UREs. However, there is lack of information on the effect of SIS UREs on the ARAIM Pfa and availability.

This paper first filters out satellite anomalies to determine the effective long-term data according to anomaly criteria. After that the BDS SIS UREs are computed and the overbound of the SIS UREs is analyzed to obtain the value of the URA. Finally, this paper analyzes the effects of the URA and URE on the ARAIM Pfa and availability. 

## 2. Statistics and Overbound of BDS SIS URE

The section calculates the BDS SIS UREs and analyzes the overbound of the SIS UREs to obtain the value of the URA. 

### 2.1. Data Preprocessing

When comparing the BDS broadcast ephemeris with the precise ephemeris to obtain the SIS UREs, it is necessary to use the useful data and remove the offsets between systems. This section filters out the outliers to obtain effective data and removes the time offset to correct the clock errors.

#### 2.1.1. Data Sources

The broadcast ephemeris file used in this study is in the RINEX V3 format and is generated by the center of the Multi-GNSS Experiment (MGEX). The precise ephemeris contains accurate satellite orbital elements and clock error information. We consider that the precise ephemeris can replace the real satellite orbital elements to calculate the SIS UREs because the precise ephemeris and the real satellite orbital elements are of the same order of magnitude, with errors of only a few centimeters; thus, the precise ephemeris is much more accurate than the broadcast ephemeris [11]. The precise ephemeris is generated by the International GNSS Service (IGS) and MGEX Analysis Center and can be obtained from a public database. 

#### 2.1.2. BDS Outlier Filter

Not all data can be statistically analyzed when calculating the SIS UREs. For example, the GPS SPS Performance Standard [12] has defined that SIS UREs must be below 4.42 times URA, and that users cannot use a broadcast ephemeris that is older than 4 h or corrupted. Therefore, the SIS UREs that satisfy any of the following conditions in this analysis are rejected [5]:

- The integrity status word is not zero, or the URA is greater than 48 m.
- The broadcast ephemeris is not within the 4 h period.
- The precise ephemeris/clock is lost.
- The SIS UREs exceed the URA by a factor of more than 4.42.

**Figure 1:**
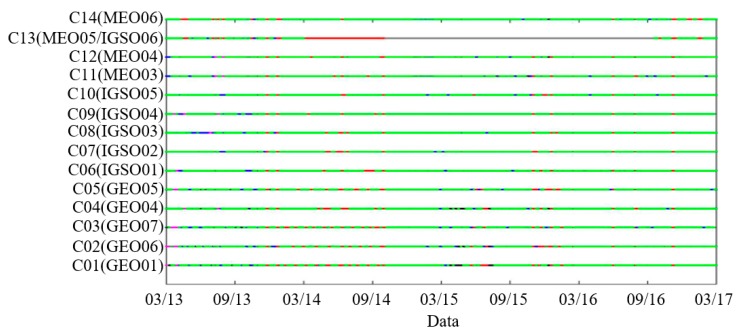
The history of the BDS constellation healthy operational status and abnormal operational status (The green dots represent a healthy operational status, and the other dots represent an abnormal operational status).

Long-term stable data are obtained by filtering out outliers according to the abovementioned criteria. Figure 1 shows the status of the 4-year BDS ephemeris. The y-axis represents the Pseudo Random Noise (PRN) and Space Vehicle Number (SVN). Green indicates that both the precise ephemeris and broadcast ephemeris are available. Red indicates that the precise ephemeris is lost but that the broadcast ephemeris is obtained. Purple indicates that the broadcast ephemeris is lost but that the precise ephemeris is available. Gray indicates that both the broadcast ephemeris and precise ephemeris are unavailable. Blue indicates that the broadcast ephemeris is corrupted. Black indicates abnormal clock errors. Satellite C13 is special among all the satellites because there are no ephemeris data nearly half the time. On 20 March 2014, the precise ephemeris of satellite C13 was missing. On 22 October 2014, the broadcast ephemeris of satellite C13 was missing. On 11 October 2016, both the broadcast ephemeris and precise ephemeris can be obtained simultaneously. The satellite MEO05 was replaced by satellite IGSO06 in January 2016, so C13 can represent both satellites. Because of the unique characteristics of C13, this satellite is excluded when calculating the anomalous probability of ephemeris data. There are 455,520 ephemeris data points for the other 13 satellites. There are 23,868 anomalous data points; thus, 5.24% of the ephemeris data are filtered out, and the anomalous data in which the precise ephemeris is lost but there is a broadcast ephemeris appears more than the other types of anomalous data.

### 2.2. Statistics of BDS SIS URE

When calculating the SIS UREs, there are four types of SIS UREs that must be considered: instantaneous SIS UREs, global average rms SIS UREs, orbit-only rms SIS UREs and worst-case SIS UREs [11]. This section mainly considers the computation of the global average rms SIS UREs, orbit-only rms SIS UREs and worst-case SIS UREs.

#### 2.2.1. Global Average rms SIS UREs, Orbit-Only rms SIS UREs and Worst-Case SIS UREs

Global average rms SIS UREs, orbit-only rms SIS UREs and worst-case SIS UREs for BDS are defined as follows [13]:

- BDS global average rms SIS UREs. There are three orbits of satellites in BDS: MEO, IGSO and GEO; satellites C01 to C05 are GEO satellites, satellites C06 to C10 are IGSO satellites, and satellites C11 to C14 are MEO satellites. Because of the differences in satellite observation geometry structure, the computational models for the SIS UREs of the three orbits are different and are derived as follows:
  (1) $$SIS\;URE_{BDS-MEO}=\sqrt{{\left(0.98R-T\right)}^{2}+\frac{1}{54}\left(A^{2}+C^{2}\right)}$$
  (2) $$SIS\;URE_{BDS-IGSO/GEO}=\sqrt{{\left(0.99R-T\right)}^{2}+\frac{1}{127}\left(A^{2}+C^{2}\right)}$$
  where *R* represents the radial errors, *A* represents the along-track errors, and *C* represents the cross-track errors. *R*, *A* and *C* are the three directions of the orbit errors. *T* represents clock errors.
- BDS orbit-only rms SIS UREs. Orbit-only rms SIS UREs are obtained by removing the clock errors according to Equations (1) and (2):
  (3) $$SIS\;URE_{only-orbit-MEO}=\sqrt{{\left(0.98R\right)}^{2}+\frac{1}{54}\left(A^{2}+C^{2}\right)}$$
  (4) $$SIS\;URE_{only-orbit-IGSO/GEO}=\sqrt{{\left(0.99R\right)}^{2}+\frac{1}{127}\left(A^{2}+C^{2}\right)}$$
- BDS worst-case SIS UREs. The worst-case SIS UREs are the maximum instantaneous SIS UREs calculated by the broadcast ephemeris. The worst-case SIS UREs play a crucial role in the SIS performance evaluation. The worst-case SIS UREs can be calculated either from the instantaneous SIS UREs or from radial errors, along-track errors, cross-track errors and clock errors. The present study uses the latter approach [14]:
  (5) $$SISURE_{worst}=\underset{|\theta |\leq \gamma }{\max}(R\cos\theta -T+\sqrt{A^{2}+C^{2}}\sin\theta )$$
  where *γ* is the latitude of the edge of satellite coverage.

#### 2.2.2. Analysis of BDS Clock Errors, Orbit Errors and SIS UREs

The time system of the BDS broadcast ephemeris is BeiDou Time (BDT), and the time system of the precise ephemeris is GPS Time (GPST); thus, the time systems must be unified when comparing the broadcast ephemeris with the precise ephemeris. Therefore, we remove the BDT-GPST bias of 14 s [10]. The broadcast ephemeris is the reference single-frequency signal B3I, maintained by the BDS military time-frequency. However, the BDS precision clock is produced by Wuhan University, calculated using dual frequency (B1I and B2I) ionosphere-free observations that use a monitoring station receiver clock as a reference [15]. When calculating the positions of satellites according to the broadcast ephemeris, there are Time Group Delays (TGDs) in signal B1I and signal B2I. The TGDsT_GD1_ and T_GD2_, which are less than one nanosecond, should be removed. In addition, the different timescales of the broadcast ephemeris and precise ephemeris cause a time offset that should be considered [16].

When calculating the orbit errors, the BDS broadcast ephemeris uses the China Geodetic Coordinate System 2000 (CGCS 2000), and the precise ephemeris uses the International Terrestrial Reference Frame (ITRF) when calculating orbit and clock errors. The differences between the CGCS 2000 and ITRF coordinate systems are ignored [17]. The precise ephemeris provides the center-of-mass coordinates of the satellites, and the broadcast ephemeris refers to the antenna phase center. However, the BDS broadcast ephemeris refers to a position close to the center-of-mass [18]. Therefore, the antenna phase center offsets do not have a significant effect and can be ignored.

**Figure 2:**
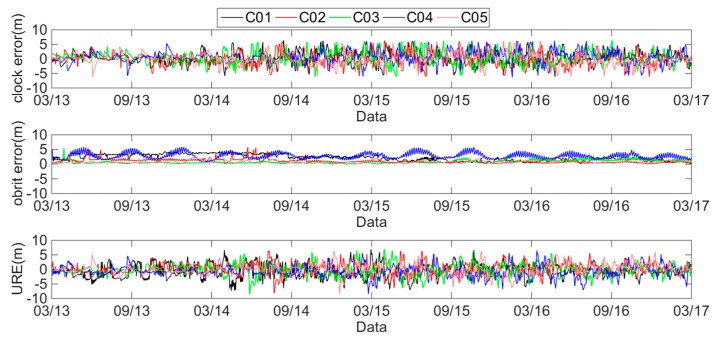
Errors of GEO satellites from 1 March 2013 to 1 March 2017.

**Figure 3:**
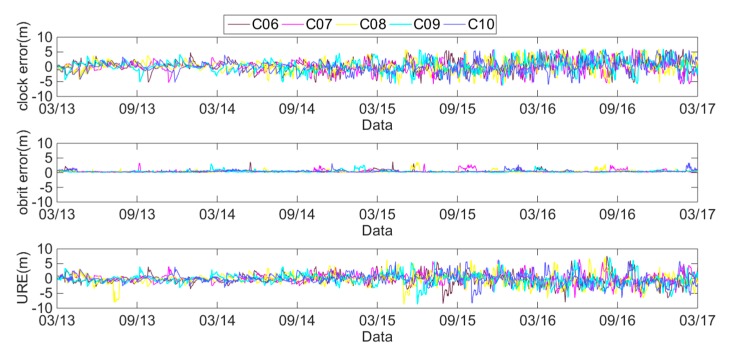
Errors of IGSO satellites from 1 March 2013 to 1 March 2017.

**Figure 4:**
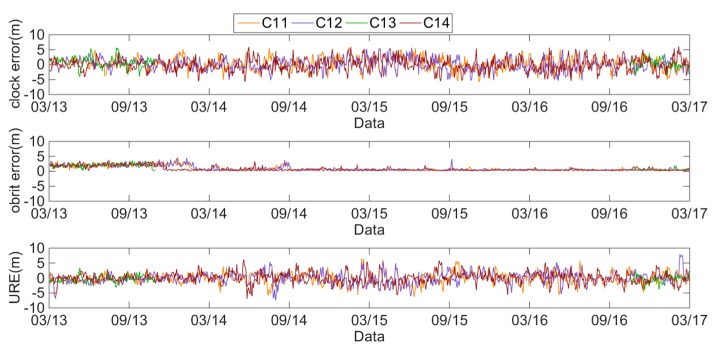
Errors of IGSO satellites from 1 March 2013 to 1 March 2017.

The daily average clock errors, orbit errors and SIS UREs of the GEO, IGSO and MEO satellites are respectively analyzed using Figure 2, Figure 3 and Figure 4.

These figures show that the clock errors of the GEO and IGSO satellites change significantly, and the maximum value of the clock errors can exceed 5 m. However, the trend of the clock errors of the MEO satellites are relatively stable. Therefore, the clock stability of the MEO satellites is better than that of the IGSO and GEO satellites. Comparing the orbit errors of GEO, IGSO and MEO satellites, it can be found that the orbit errors of the GEO satellites are the worst, especially the C04 satellite. The orbit errors of the C04 satellite have a value of 2–6 m. Notably from April 2013 to August 2014, the orbit errors of the C01 satellite are 3–4 m, but most of the rest of the time, the orbit errors are under 1 m. The orbit errors of the IGSO satellites have the best performance, with most of the orbit errors under 0.5 m. However, some orbit errors may exceed 4 m. From March 2013 to January 2014, the orbit errors of the MEO satellites approached 4 m, but after January 2014, they were almost under 0.5 m, with only a few orbit errors exceeding 2 m. Therefore, the BDS orbit errors have differences because of the different types of BDS satellites.

### 2.3. Overbound BDS SIS UREs

SIS UREs are usually assumed to be overbounded by a normal distribution with a standard deviation represented by the URA. The URA is an estimate of the UREs caused by the satellite section, which requires overbound SIS UREs with a probability of 68% [19]. The URA is used to describe the accuracy of satellite space signals and is characterized by the User Range Accuracy Index (URAI). The broadcast URA is a relatively conservative estimate of the standard deviation of SIS UREs and is mainly used for integrity monitoring. 

The BDS broadcast ephemeris contains a URAI with a value of 2. When the URAI is converted to a URA, the URA is a value range instead of a fixed value. According to the CSNO (2016) BeiDou navigation satellite system signal in space interface control document [20], when 0 < N ≤ 6, URA = 2^N/2+1^. Therefore, when N = 0, 0 < URA ≤ 2.4. Because the broadcast URA is 2 m, the upper URA in the interface control document is 2.4 m.

**Figure 5:**
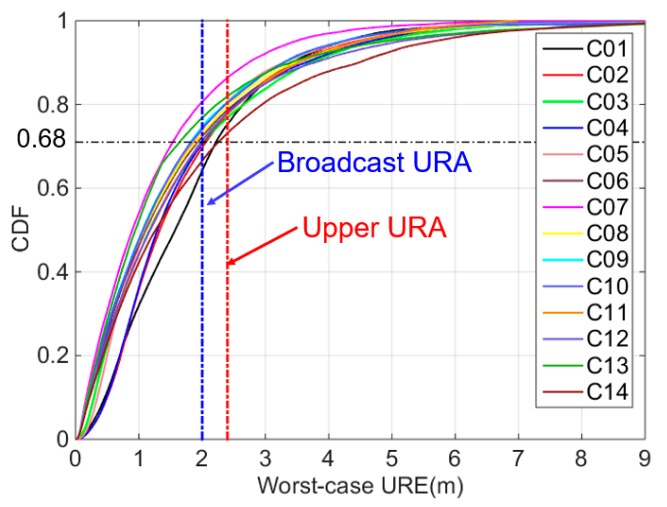
Cumulative distribution functions of the worst-case UREs for each BDS satellite (The blue dashed line indicates the broadcast URA of 2 m, the red dashed line indicates the upper URA of 2.4 m, and the black dashed line indicates the 68% overbound).

The cumulative distribution functions of the worst-case UREs are shown in Figure 5. The blue dashed line shows that the URA is 2 m, and the red dashed line shows that the URA is 2.4 m. The black dashed line denotes the 68% overbounds. The required overbound of satellite C01 is the largest and that of satellite C07 is the smallest. When using a broadcast URA of 2 m, the overbound of satellite C01 is 61.47%, which is below 68% and that of satellite C07 is 88.02%. When using the upper URA of 2.4 m, the overbound of satellite C01 is 78.68%, and that of satellite C07 is 92.62%.

Table 1 lists the 68% overbound worst-case UREs. Satellite C01 has the largest 68% overbound, and satellite C07 has the smallest. Therefore, the upper URA of 2.4 m can overbound the worst-case UREs of the 14 BDS satellites. However, the broadcast URA of 2 m cannot overbound the SIS UREs of all BDS satellites such as satellites C01, C02, C03, and C14. Therefore, the broadcast URA is not sufficiently conservative, which is inconsistent with the performance of the worst-case UREs. 

## 3. Effect of BDS URA on ARAIM

According to the previous section, the BDS broadcast URA is not consistent with the historical performance of SIS UREs, but the URA of 2.4 m can satisfy the requirement. As a result, this section analyzes the Pfa and ARAIM availability when using URAs of 2.4 m and 2 m, respectively.

### 3.1. Effect of BDS URA on Pfa

The ARAIM algorithm relies on the ground monitoring network to observe the state of the satellite and provide this information to aircraft in the form of an ISM to meet the requirements of system integrity and continuity [21]. ISM parameters (either online or offline) can be divided into two types: fault probability and nominal errors. The first group of parameters includes the probability of a satellite fault *P_sat_* and the probability of a constellation fault *P_const_*. The second group of parameters includes the nominal SISE, which provides the user with three parameters used to overbound nominal pseudorange errors: *σ_URE_*, *σ_URA_* and the maximum nominal bias *b_nom_* [22]. The values of *σ_URE_* and *σ_URA_* have an effect on the calculation of Pfa.

According to the ARAIM solution separation [23], the coefficient of Pfa allocated to the vertical and horizontal directions are derived as follows:

(6) $$K_{fa,q}=\frac{T_{k,q}}{\sigma_{ss,q}{}^{\left(k\right)}}=Q^{-1}(\frac{P_{FA\_HOR}}{4N_{fault\_\mathrm{mod}es}})\;q\;=\;1,\;2$$

(7) $$K_{fa,q}=\frac{T_{k,q}}{\sigma_{ss,q}{}^{\left(k\right)}}=Q^{-1}(\frac{P_{FA\_VERT}}{2N_{fault\_\mathrm{mod}es}})\;q\;=\;3$$

where *σ_ss,q_^(k)^* is the standard deviation of the difference between the all-in-view and the fault tolerant position solutions. *T_k,q_* is the threshold of the solution separation test. *P_FA_HOR_* is the horizontal Pfa, and *P_FA_VERT_* is the vertical Pfa. *k* represents the *k*-th failure mode. $N_{fault\_\mathrm{mod}es}$ is the number of fault modes. The indices *q* = 1, 2 and 3 designate the east, north and up components, respectively. The standard deviation *σ_ss,q_^(k)^* is calculated by:

(8) $$\sigma_{ss,q}{}^{(k)2}=(S_{q}^{\left(k\right)}-S_{q}^{\left(0\right)})C_{acc}{\left(S_{q}^{\left(k\right)}-S_{q}^{\left(0\right)}\right)}^{T}$$

(9) $$S^{\left(k\right)}={\left(G^{T}W^{\left(k\right)}G\right)}^{-1}G^{T}W^{\left(k\right)}$$

where *G* is the geometry matrix in terms of the east, north, and up components with a clock component for each constellation. *W* is the weighting matrix. *C_acc_* is the pseudorange error diagonal covariance matrix for accuracy and continuity:

(10) $$C_{acc}=\sigma_{URE,i}^{2}+\sigma_{tropo,i}^{2}+\sigma_{user,i}^{2}$$

(11) $$\begin{array}{l} W^{\left(k\right)}=W^{\left(k\right)}(j,j)=0\;if\;j\;is\;in\;idx_{k}\\ W^{\left(k\right)}=W^{\left(k\right)}(j,j)=C_{\mathrm{int}}^{-1}(j,j)\;otherwise \end{array}$$

where *j* is the index of the constellation, and *idx_k_* is the indices of the satellites included in fault subset k. $C_{\mathrm{int}}(j,j)$ is the pseudorange error diagonal covariance matrix for integrity:

(12) $$C_{\mathrm{int}}=\sigma_{URA,i}^{2}+\sigma_{tropo,i}^{2}+\sigma_{user,i}^{2}$$

where *σ_tropo,i_* and *σ_user,i_* denote the tropospheric delays and user errors, respectively. They are related to the elevation angle: the calculation model for *σ_tropo,i_* is [1]:

(13) $$\sigma_{tropo}=0.12\times \frac{1.001}{\sqrt{0.002001+{\left(\sin(\frac{\theta \pi }{180})\right)}^{2}}}$$

The calculation model for *σ_user,i_* is [1]:

(14) $$\begin{array}{l} \sigma_{user,i}=\sqrt{\frac{f_{B1I}^{4}+f_{B2I}^{4}}{\left(f_{B1I}^{2}-f_{B2I}^{2}\right)}}\times \sqrt{{\left(\sigma_{MP}\right)}^{2}+{\left(\sigma_{Noise}\right)}^{2}}\\ \sigma_{MP}(\theta )=0.13+0.53\exp(-\theta /10)\\ \sigma_{Noise}(\theta )=0.15+0.43\exp(-\theta /6.9) \end{array}$$

where θ is the elevation angle in degrees, $f_{B1I}$ and $f_{B2I}$ are the frequencies of signal B1 and signal B2, $\sigma_{MP}$ is the multipath error, and $\sigma_{Noise}$ is the receiver noise.

According to the above formula, the Pfa can be derived as follows:

(15) $$\begin{array}{l} P_{fa}=2\sum_{q=1}^{3}\sum_{k=0}^{N_{fault\_\mathrm{mod}es}}Q(K_{fa,q}^{\left(k\right)})=2\sum_{q=1}^{3}\sum_{k=0}^{N_{fault\_\mathrm{mod}es}}Q(\frac{T_{k,q}}{\sigma_{ss,q}^{\left(k\right)}})\\ =2\sum_{q=1}^{3}\sum_{k=0}^{N_{fault\_\mathrm{mod}es}}Q(\frac{T_{k,q}}{(S_{q}^{\left(k\right)}-S_{q}^{\left(0\right)})(\sigma_{URE,i}^{2}+\sigma_{tropo,i}^{2}+\sigma_{user,i}^{2}){\left(S_{q}^{\left(k\right)}-S_{q}^{\left(0\right)}\right)}^{T}}) \end{array}$$

where *Q* represents the cumulative distribution function of a zero mean unit Gaussian distribution.

**Figure 6:**
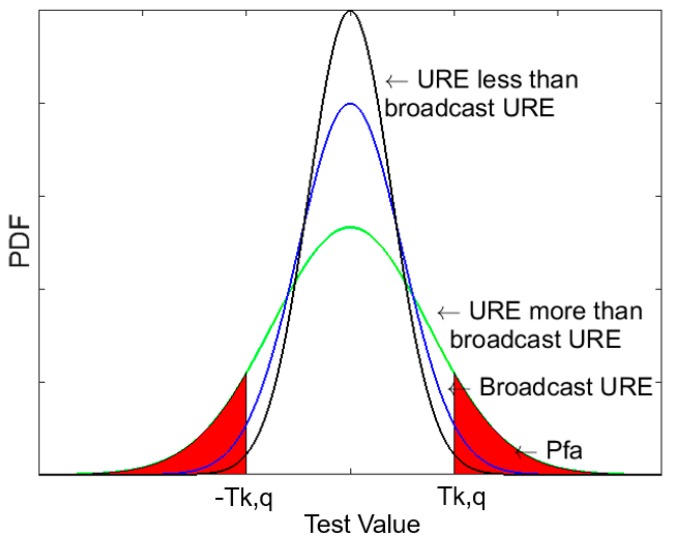
Probability density function of the fault detection test.

To verify the relationship between the *σ_URA_*/*σ_URE_* and Pfa, we choose 14 BDS satellites for the ARAIM simulation. If only single satellite faults and single constellation faults are considered, then the total number of *N_fault_modes_* = 15. When the solution separation test is conducted, the east, north and up components must be considered; thus, the total fault detection quantity is 45. Because the number of tests is enough, it is common to find at least one test for which *T_k,q_* is relatively small. The *T_k,q_* is the threshold of the solution separation test. If the solution exceeds the threshold, the result of the detection test can be considered a false alert. In this case, the *P_fa,q_^(k)^* changes as *σ_URE_* varies. As shown in Figure 6, the change in *σ_URE_* causes the probability density function to change, and the Pfa changes if the threshold *T_k,q_* is constant. The black and green lines respectively represent the computed UREs that cause the computed *σ_URE_* to be less than the broadcast *σ_URE_* and greater than the broadcast *σ_URE_*, the blue line represents the broadcast URE, and the red region represents the Pfa. When the threshold *T_k,q_* is constant, the red area corresponding to the black line is smaller than that corresponding to the blue line which means the Pfa decreases. For the same reason, the Pfa corresponding to the green line increases.

**Figure 7:**
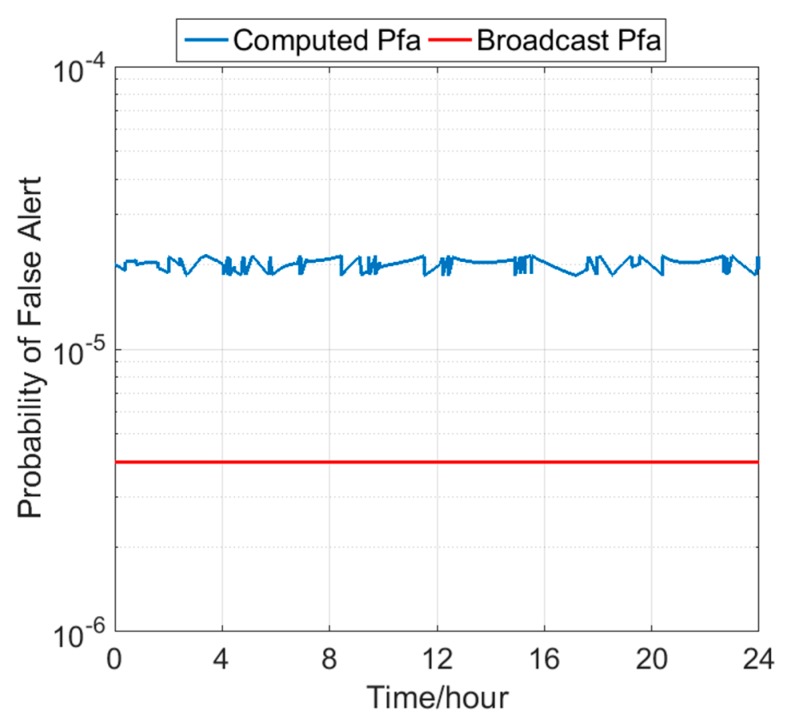
Comparison between the Pfa in the ISM and the computed Pfa on 1 March 2017.

According to the previous section, the broadcast URA of 2 m cannot meet the accuracy requirement of the observed historical SIS URE performance from 1 March 2013 to 1 March 2017, but the upper URA of 2.4 m can satisfy the 68% overbound of the worst-case UREs. Therefore, we use the computed *σ_URA_* of 2.4 m. The EU-U.S. Cooperation on Satellite Navigation Working Group C-ARAIM Technical Subgroup Milestone 3 Report defined that the URE was set to be two thirds of the URA [24] The value of *σ_URE_* is set to 1.6 m. The *σ_URA_* and *σ_URE_* are used to evaluate the Pfa and compare it with the Pfa ISM. In Figure 7, the red line represents the Pfa in the ISM of 4 × 10^−6^, and the blue line represents the computed Pfa, whose value is approximately 2 × 10^−5^. The computed Pfa exceeds the Pfa in the ISM by an order of magnitude, which results in the degradation of the continuity. 

According to the previous section, the performances of the GEO, IGSO and MEO satellites vary. SIS UREs of different satellites can be considered to be uncorrelated in ARAIM [6]. Therefore, the standard deviations *σ_URE_* of satellites C01, C06 and C14 are selected to consider the change in the Pfa in one day. The SIS UREs are selected to calculate the Pfa on 1 March 2017. The time interval is 1 s, and the total number of samples is 86,400. We take the sample value per minute to obtain *σ_URE_*.

**Figure 8:**
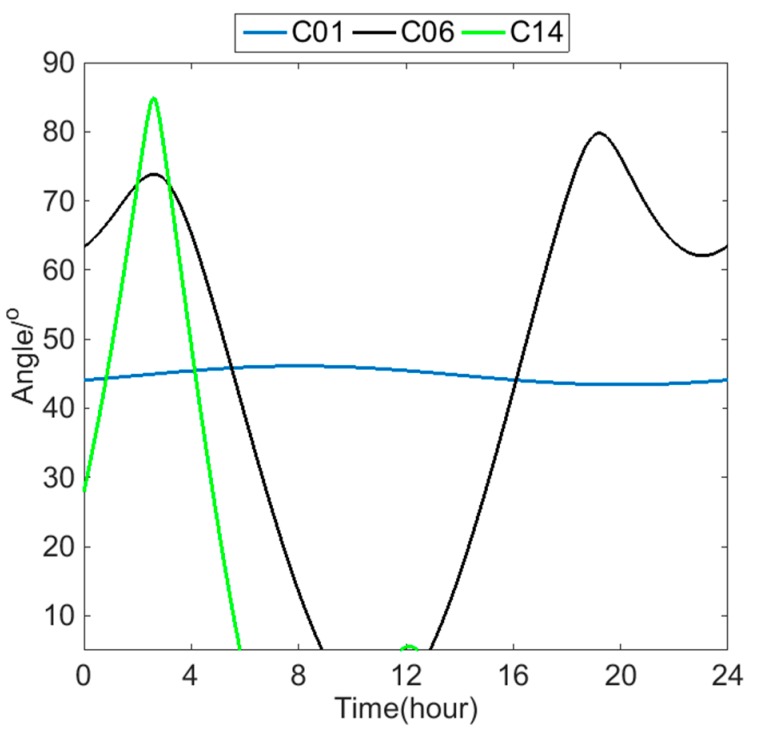
Daily elevation variety of satellites C01, C06 and C14 as seen from the receiver at Wuhan University.

Because a single satellite is considered, each satellite undergoes a period during each day when it is not within the field of view of the receiver at Wuhan University. Pfa changes only within the field of view of the satellite are considered, which means that changes in the satellite elevation angle must be considered. The mask angle is set to 5°, and the results are shown in Figure 8. The blue line is the elevation angle of satellite C01, which is 43.4°–46.1° because the relative position between the GEO satellites and ground remains unchanged. Therefore, the GEO satellites are readily visible. The black line is the elevation angle of satellite C06, which is 5°–79.7°. The elevation angle is below the defilade angle for the 4 h between 9 and 13 h and is thus invisible. The elevation transformation of satellite C06 is approximately symmetrical because the orbit of the IGSO satellite is an “8” shaped orbit, and the 24 h trajectory under the satellite oscillates between north and south within the service area. The green line is the elevation angle of satellite C14, which is 5°–84.9°. The satellite is visible only between 0 and 6 h and between 11:50 and 12:50 because the 24 h trajectory under the satellite moves globally.

**Figure 9:**
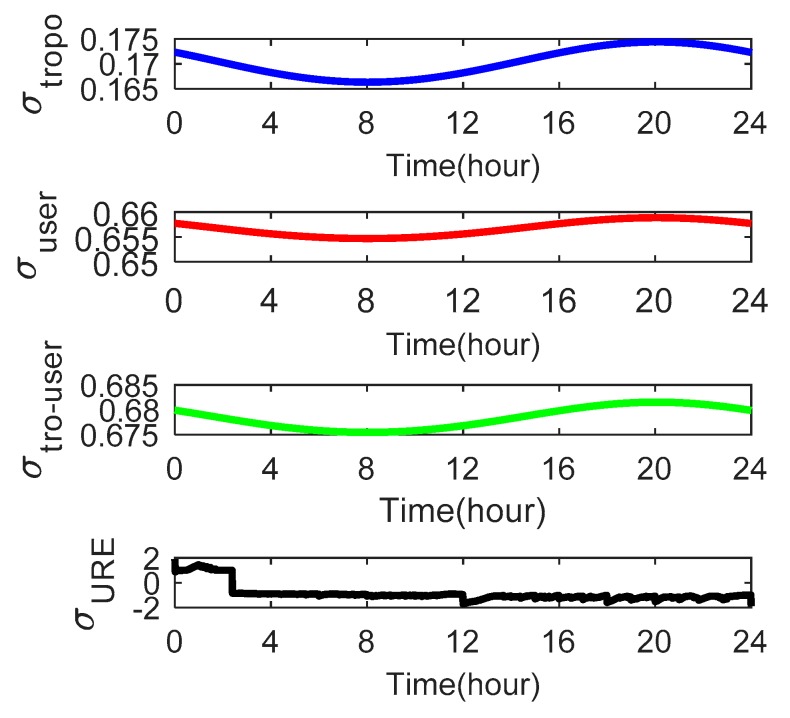
The tropospheric delays *σ_tropo,i_*, user errors *σ_user,i_* and *σ_URE_* of satellite C01 over one day as seen from the receiver at Wuhan University.

**Figure 10:**
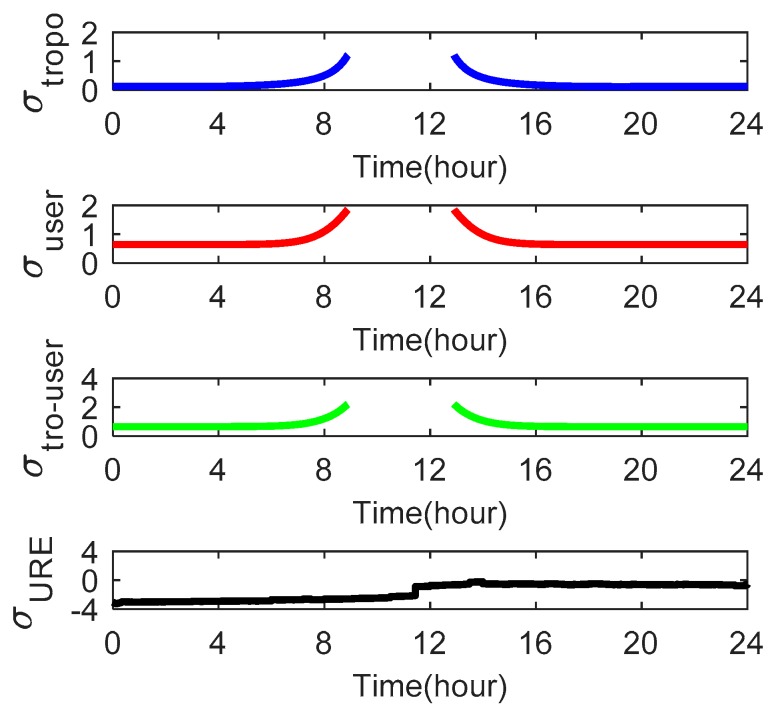
The tropospheric delays *σ_tropo,i_*, user errors *σ_user,i_* and *σ_URE_* of satellite C06 over one day as seen from the receiver at Wuhan University.

**Figure 11:**
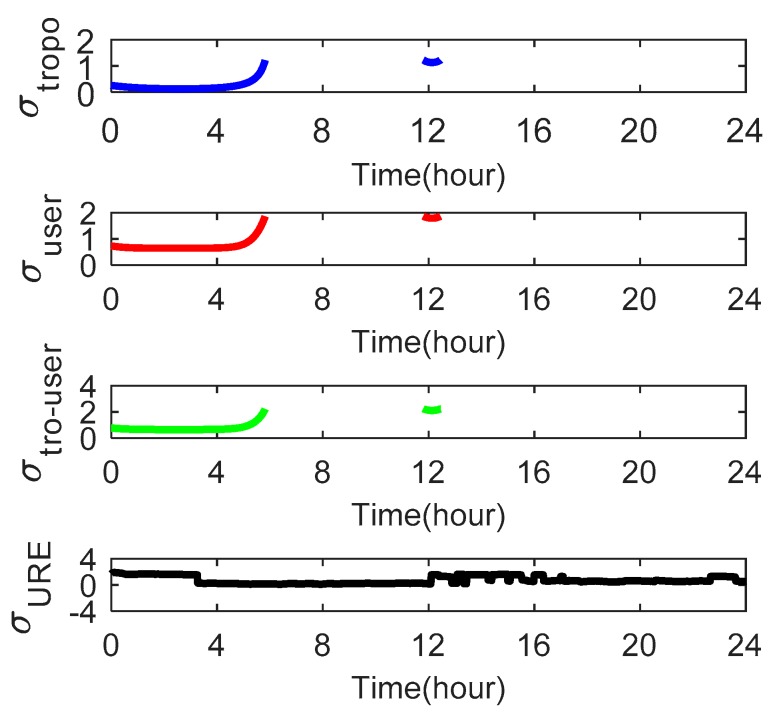
The tropospheric delays *σ_tropo,i_*, user errors *σ_user,i_* and *σ_URE_* of satellite C14 over one day as seen from the receiver at Wuhan University.

As the satellite elevation angle changes, the tropospheric delays *σ_tropo,i_* and user errors *σ_user,i_* also change. Figure 9, Figure 10 and Figure 11 show these parameters for satellites C01, C06 and C14, respectively. We use the Beijing Time in one day as the x-axis. 

Figure 9 shows that satellite C01 has a *σ_tropo,i_* of 0.165–0.175 and a *σ_user,i_* of 0.65–0.66 m. The value of *σ_URE_* is greater than *σ_tropo,i_* and *σ_user,i_* at all times for satellite C01. Figure 10 shows that satellite C06 has a *σ_tropo,i_* of 0.13–1.21 m. At 0–5 h and 17–24 h, the value of *σ_tropo,i_* changes slowly and approximates a straight line because the elevation angle in these two periods is large. For the same reason, the user errors *σ_user,i_* approximate a straight line at 0–6 h and 16–24 h. Figure 11 shows that satellite C14 has a *σ_tropo,i_* of 0.12–1.226 m and a *σ_user,i_* of 0.64–1.85 m. The values of *σ_tropo,i_* and *σ_user,i_* of satellites C06 and C14 are similar but greater than those of C01 because the elevation angle of the GEO satellites is larger and substantially unchanged relative to the receiver. In addition, the value of *σ_user,i_* for all three satellites is greater than *σ_tropo,i_*, which indicates that the effect of *σ_user,i_* on the Pfa is greater than the effect of *σ_tropo,i_*.

**Figure 12:**
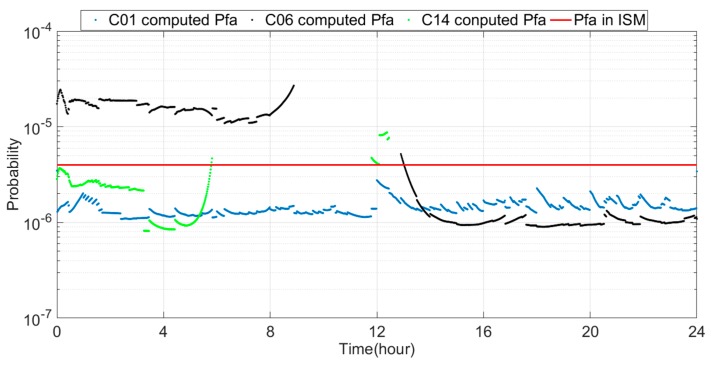
Computed Pfa of satellites C01, C06 and C14 per minute.

The changes in *σ_tropo,i_*, *σ_user,i_* and *σ_URE_* on 1 March 2017, are used to derive the Pfa. The results are shown in Figure 12 (X-axis is the Beijing Time in one day): satellite C01 has a Pfa of 1.09 × 10^−6^–2.76 × 10^−6^, satellite C06 has a Pfa of 9.38 × 10^−7^–2.70 × 10^−5^, and satellite C14 has a Pfa of 8.45 × 10^−7^–8.73 × 10^−6^. We call the Pfa calculated by the computed *σ_URE_* the computed Pfa. The computed Pfa of satellite C01 is less than the Pfa in the ISM because the computed *σ_URE_* is less than the broadcast *σ_URE_*. The degradation of Pfa may reach 62.5%. Approximately 55% of the computed Pfa values of satellite C06 are less than the Pfa in the ISM and the largest drop reaches 76.6% because the computed *σ_URE_* is close to 0 m. The other computed Pfa values are greater than the Pfa in the ISM because the computed *σ_URE_* is close to 4 m. Most of the computed Pfa values of satellite C14 are smaller than the Pfa in the ISM and the greatest drop reaches 78.9%. The computed Pfa drops sharply at approximately 3 h because the computed *σ_URE_* decreases to 0 m. The computed Pfa rapidly increases and ultimately exceeds the Pfa in the ISM at 3–6 h, which is related to *σ_tropo,i_* and *σ_user,i_*. Therefore, the Pfa may be mainly affected by *σ_URE_* but also partly affected by *σ_tropo,i_* and *σ_user,i_*. In the case of a small *σ_URE_*, it is possible that *σ_tropo,i_* and *σ_user,i_* strongly affect the Pfa. 

### 3.2. Effect of BDS URA on ARAIM Availability

In the section, the computed *σ_URA_* of 2.4 m and computed *σ_URE_* of 1.6 m are used to evaluate the availability. GPS satellites and 14 BDS satellites are used to perform an ARAIM simulation. The parameters in the ISM are set as shown in Table 2. 

**Figure 13:**
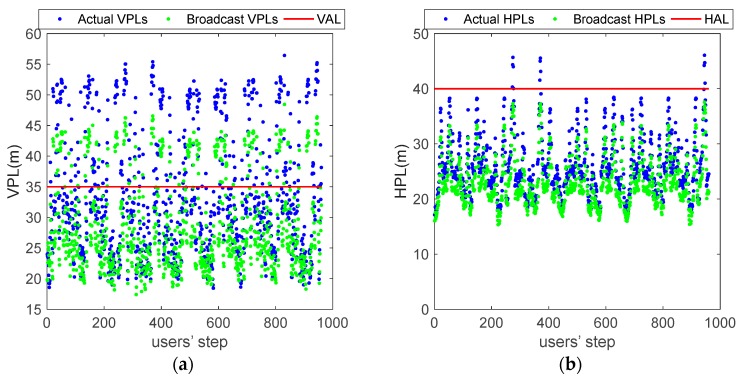
PLs for the computed *σ_URE_*/*σ_URA_* and the broadcast *σ_URE_*/*σ_URA_*. (**a**)VPLs; (**b**) HPLs.

Figure 13 shows the Vertical Protection Levels (VPLs) and Horizontal Protection Levels (HPLs) with simulation points for the computed *σ_URA_*/*σ_URE_* and the broadcast *σ_URA_*/*σ_URE_* (960 data points in each figure). The VPLs/HPLs calculated by the computed *σ_URA_*/*σ_URE_* are referred to as the actual VPLs/HPLs and the VPLs/HPLs calculated by the broadcast *σ_URA_*/*σ_URE_* are referred to as the broadcast VPLs/HPLs. Blue points represent the actual VPLs/HPLs, green points represent the broadcast VPLs/HPLs, and red lines represent the alert values for the HPL and VPL. The actual VPLs and HPLs are greater than the broadcast values. There are 340 actual VPLs and 209 broadcast VPLs exceeding the Vertical Alert limit (VAL); only 13 actual HPLs exceed the Horizontal Alert limit (HAL), and all broadcast HPLs are below the HAL. Therefore, in some cases, the Protection Levels (PLs) calculated by the computed *σ_URA_*/*σ_URE_* exceed the Alert Level (AL), but those calculated by the broadcast *σ_URA_*/*σ_URE_* are below the AL, which may lead to missing alert. The increase in the number of missing alerts may lead to integrity degradation.

**Figure 14:**
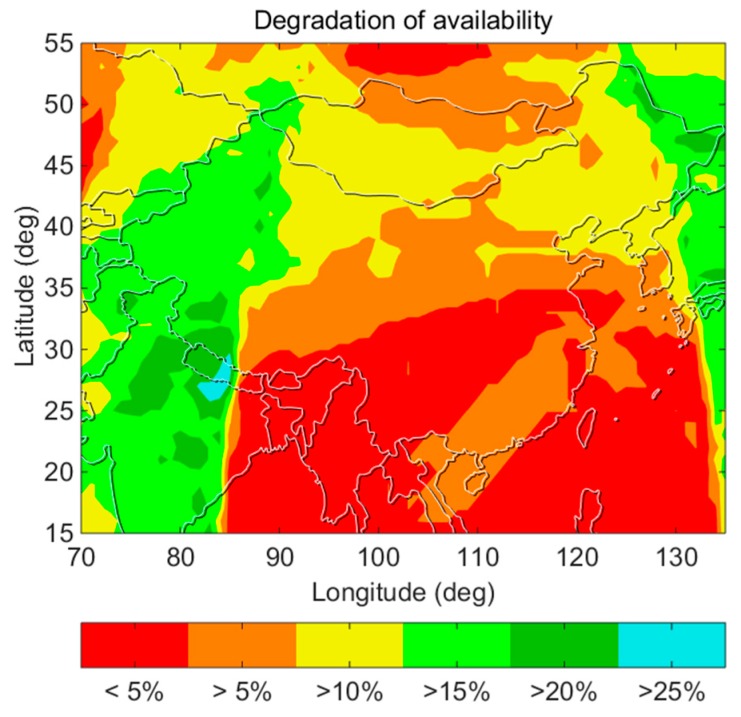
Degradation of the availability in various regions of China.

Figure 14 shows the degradation of the availability for the computed *σ_URA_*/*σ_URE_* relative to the broadcast *σ_URA_*/*σ_URE_*. The greatest degradation of the availability is approximately 25%. The degradation of the availability for the southern region in China is within 10% and is smaller than that for the northern region because the trajectories of the five GEO satellites are fixed points on the equator. For these five satellites, there is a relatively high elevation angle at low latitudes, while the elevation angle at high latitudes is relatively low. The Geometric Dilution of Precision (GDOP) of the GEO satellite changes with the variation in the latitude, the value of the GDOP at low latitudes is smaller than that at high latitudes [25]. 

## 4. Conclusions

To study the effect of the computed *σ_URA_*/*σ_URE_* on the Pfa and availability, we obtained the corresponding SIS UREs by comparing the broadcast ephemeris with the precise ephemeris from 1 March 2013 to 1 March 2017. For the broadcast ephemeris and precise ephemeris, 23,868 outliers were statistically removed to obtain useful data. Then, the overbound of the SIS UREs is analyzed. The result shows that the broadcast URA of 2 m does not satisfy the performance requirements of observed historical SIS UREs and is slightly optimistic, but the upper URA of 2.4 m is more suitable for users. When using the computed *σ_URA_* of 2.4 m and *σ_URE_* of 1.6 m to calculate the Pfa, the computed Pfa may exceed the Pfa in the ISM by an order of magnitude. An increase in the Pfa will lead to a degradation of the continuity. Considering the effect of *σ_URE_* on the Pfa for GEO, IGSO and MEO satellites, the three types of satellites have different effects on the Pfa. The result shows that the computed Pfa values of satellite C01 are less than the Pfa in ISM, approximately 55% of the computed Pfa values of satellite C06 are less than the Pfa in ISM and most of the computed Pfa values of C14 are less than the Pfa in the ISM. The effects of *σ_tropo,i_* and *σ_user,i_* are less than that of *σ_URE_*, but they may greatly affect the Pfa in the case of a small *σ_URE_*. The PLs calculated by the computed *σ_URA_*/*σ_URE_* are larger than those calculated by the broadcast *σ_URA_*/*σ_URE_*, sometimes even beyond the AL which can lead to missing alerts. The integrity will degrade because of increases in the number of missing alerts. The availability of the computed *σ_URA_*/*σ_URE_* is less than that of the broadcast *σ_URA_*/*σ_URE_*. The greatest degradation of the availability is approximately 25%. The degradation of the availability for the southern region in China is less than that for the northern region.

## Figures and Tables

**Table 1:** Worst-case UREs: 68%.

PRN	68% Overbounds of Worst-Case UREs(m)
C01	2.208
C02	2.027
C03	2.069
C04	1.878
C05	1.722
C06	1.777
C07	1.567
C08	1.743
C09	1.688
C10	1.689
C11	1.844
C12	1.843
C13	1.629
C14	2.135

**Table 2:** ISM basic parameter settings.

Parameter	Setting
GPS URA/URE	1/0.667
BDS URA/URE	2/1.333 (broadcast) 2.4/1.6 (computed)
*b_nom_*	0.75
*P_sat,GPS_/P_sat,BDS_*	10^−5^
*P_const,GPS_/P_const,BDS_*	10^−4^
